# Association of a multiple-step action with cervical lymph node yield of oral cancer patients in an Asian country

**DOI:** 10.1186/s12903-021-01389-3

**Published:** 2021-01-13

**Authors:** Ching-Chieh Yang, Bor-Hwang Kang, Wen-Shan Liu, Chun-Hao Yin, Ching-Chih Lee

**Affiliations:** 1 Department of Radiation Oncology, Chi-Mei Medical Center, Tainan, Taiwan; 2 Department of Pharmacy, Chia-Nan University of Pharmacy and Science, Tainan, Taiwan; 3 Department of Otolaryngology, Head and Neck Surgery, Kaohsiung Veterans General Hospital, Kaohsiung, Taiwan; 4 School of Medicine, National Defense Medical Center, Taipei, Taiwan; 5 Department of Pharmacy, Tajen University, Pingtung, Taiwan; 6 Department of Radiation Oncology, Kaohsiung Veterans General Hospital, Kaohsiung, Taiwan; 7 Department of Medical Education and Research, Kaohsiung Veterans General Hospital, Kaohsiung, Taiwan; 8 Institute of Hospital and Health Care Administration, National Yang-Ming University, Taipei, Taiwan

**Keywords:** Oral cancer, Neck dissection, Lymph node yield, Quality, Recurrence

## Abstract

**Background:**

High quality lymph node (LN) yield could increase survival, however strategies to improve LN yield have been seldom reported. This study aimed to assess the multiple-step action to promote quality of neck dissection in oral cancer.

**Methods:**

A total of 400 patients with oral cancer who underwent primary tumor resection and neck dissection, including elective and radical neck dissection, were recruited after propensity score matching by clinical T and N categories between January 2009 and September 2018. Patients were treated by two independent departments in our institute. A multiple-step action was initiated in October 2015 in one department, and another department was as a control group. The impact of multiple-step action on LN yield and regional recurrence were analyzed using multivariate analysis and difference-in-differences (DID) linear regression analysis.

**Results:**

The mean patient age was 55.2 + 11.1 years, and 92% were male. A total of 180 (45%) patients had T3-4 disease, and 129 (32%) patients had N2-3 disease. The multivariate linear regression and DID analyses revealed that multiple-step action had a positive effect on LN yield. A net improvement of LN yield with a coefficient of 13.78 (*p* < 0.001) after launching multiple-step action (since October 2015) was observed. A borderline protective effect of multiple-step action for cN0 patients with a reduced regional recurrence rate of 11.6% (*p* = 0.072) through DID analysis was noted.

**Conclusions:**

Multiple-step action was associated with increased LN yield and decreased regional recurrence in patients with oral cancer. The observed activity may promote surgeons to improve the quality of neck dissections, is feasible, and could be applied to a widespread patient population.

## Background

Oral cancer is still one of the most common and lethal tumors worldwide [1]. Despite great advances in multimodal therapy such as modern radiation technique, targeted therapy, immunotherapy, gene therapy and so on, treatments for oral cancer remain associated with high recurrence and rates of metastasis [2–4]. Currently, wide resection of the primary tumor with neck dissection is the cornerstone of treatment for patients with neck metastasis or a high rate of occult neck metastasis [5]. Since patients with oral cancer may have a worse prognosis upon recurrence, it is important to perform wide resection of the primary tumor with a clear margin [6]. Recently, a high quality neck dissection with adequate retrieval of lymph node (LN) was introduced and implemented in an effort to improve outcomes in different tumor types, including oral cancer [7–9].

Patients with inadequate LN harvests may experience stage migration and subsequent underestimation of disease severity [10]. Previously published literature has outlined the positive association between LN yield in neck dissection and overall survival rates [11, 12]. Ebrahimi et al. reported that a LN yield < 18 was associated with worse 5-year overall survival (hazard ratio [HR], 2.0; 95% confidence interval [CI], 1.1–3.6), disease-specific survival (HR, 2.2; 95% CI 1.1–4.5), and disease-free survival (HR, 1.7; 95% CI 1.1–2.8). Thus, LN yield ≥ 18 has been proposed as a cut-off point for adequate neck dissection. A cut-off value of 15 for elective neck dissection (END) and 25 for radical neck dissection (RND) have also been suggested [13]. However, there is no efficient intervention style to improve the quality of neck dissection.

The aim of this study was to assess the impact of multiple-step action to promote LN yield in oral cancer surgery. The change in LN yield in neck dissection among two different departments taken at different times in our institute were examined. In order to improve LN yield quality of neck dissection, a multiple-step action was initiated in October 2015 in one department, and another department was as a control group. The impact of multiple-step action on LN yield and regional recurrence were analyzed using multivariate analysis and difference-in-differences (DID) linear regression analysis [14–16].

## Methods

### Patient demographics and database

**Fig. 1:**
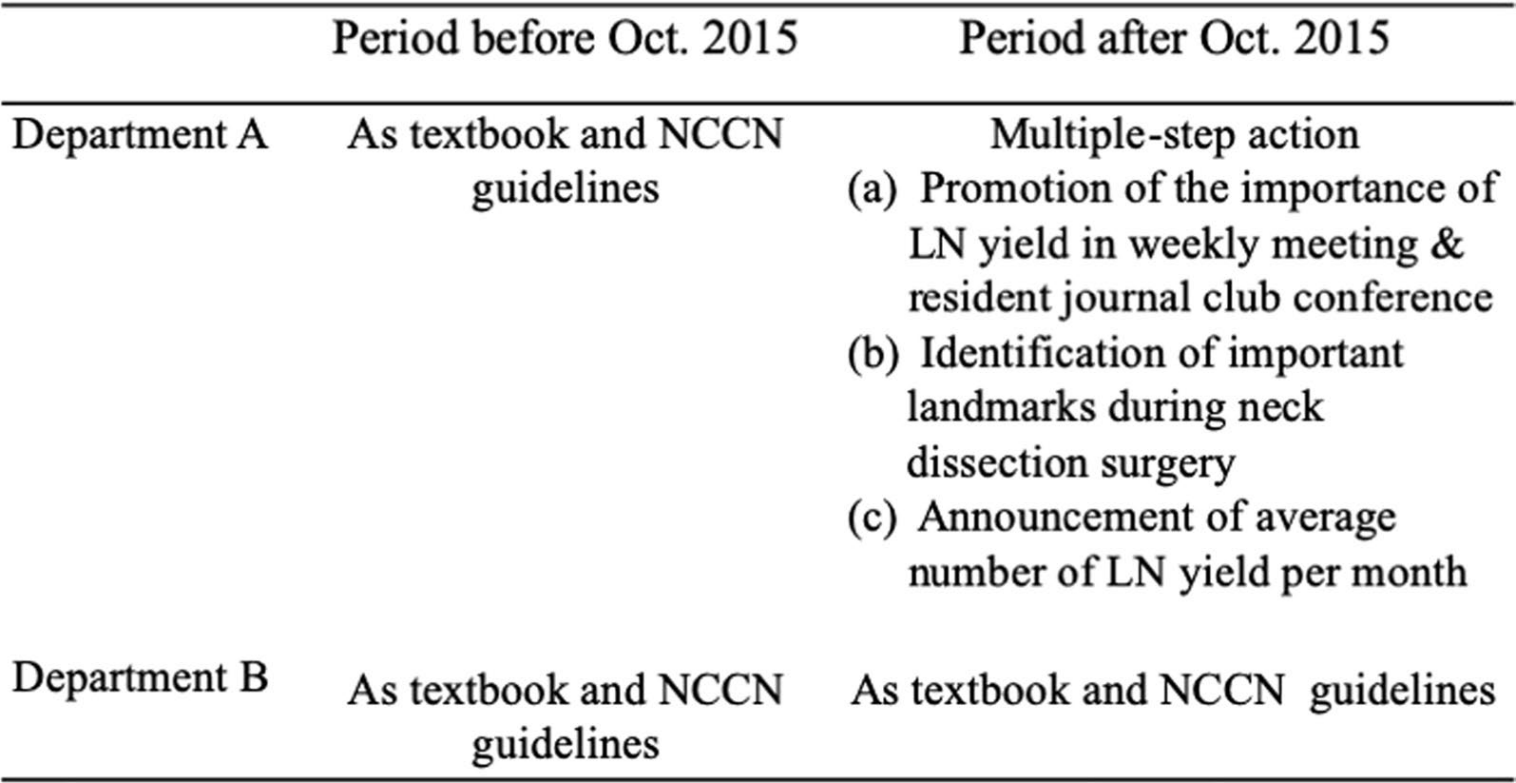
Policy change or intervention in different groups and periods

Newly diagnosed patients with oral squamous cell carcinoma treated with resection of the primary oral tumor and neck dissection with or without adjuvant therapy were identified from our hospital’s Cancer Registry Database between 2009 and 2018, retrospectively. Patients without complete treatment, electronic medical records, regular follow up, previous cancer treatment and distant metastasis upon diagnosis were excluded from the study. Neck dissection included END for clinical N0 disease and RND for clinical N1-3 disease. Patients with bilateral neck dissection and those whose procedure retrieved an LN < 10 were also excluded. The variables collected from the Cancer Registry Database included patient demographic data such as age, gender, tumor status (e.g., clinico-pathological TNM stage), and pathological risk features (e.g., margin status, tumor differentiation, perineural invasion, lympho-vascular permeation, number of retrieved LNs, number of positive LNs, extranodal extension, adjuvant treatment modality, radiation dose, and chemotherapy regimen). All staging was performed according to the American Joint Committee on Cancer (AJCC) cancer staging (7th edition). Patients with oral cancer were treated in two departments (department A and B) in our institution. Initially, LN yield was higher in department B. In order to improve quality metrics in head and neck cancer, a multiple-step action to improve cervical LN number was implemented in October 2015. As shown in Fig. 1, the study was categorized into two stages: period 1 (before October 2015), and period 2 (after October 2015). In period 1, the operative procedure for primary tumor resection and neck dissection was performed as per guidelines and/or published literature in department A and department B, respectively [5, 17]. In period 2, a non-compulsory multiple-step action plan was implemented in department A. This action plan included: (1) promotion of the importance of survival rates and LN yield in head and neck cancer in weekly meetings, and resident journal club conferences; (2) encouraging surgeons to identify important landmarks (e.g., cervical ansa, internal jugular vein, carotid artery, spinal accessory nerve, splenius capitus, levator scapula, anterior scalene muscle, and transverse cervical artery during neck dissection); (3) announcing the average number of LN yield each month, however, individual data related to the patients or surgeons was not disclosed.

Between 2009 and 2018, a total of 469 newly diagnosed patients with oral cancer who received wide resection and neck dissection were recruited. In order to reduce selection bias, propensity score matching with clinical tumor category and node category between the two departments were performed. The hypothesis was that the target group surgeons’ behavior response to the multiple-step action plan could improve LN yield. For regional recurrence, a concept of regional recurrence density (the proportion of regional recurrence among those treated with neck dissection during a specific period) was used. This method could provide a comparison of regional recurrence between different groups by time period.

### Statistical analysis

The distribution of patient, tumor, and treatment characteristics were compared between groups. Categorical variables were analyzed using the Pearson chi-square or Fisher exact test, and continuous variables were compared using a one-way analysis of variance. Comparisons between before and after changes for each outcome in the treatment group (defined as patients treated in department A) to before and after changes in the control group (defined as patients treated in department B) were performed. LN yield differences and regional recurrence between the 2 time periods, and groups was analyzed with a two-sample t-test and Pearson’s chi-square test/Fisher’s exact test, respectively. Multivariate regression was performed. Furthermore, DID comparison of LN yield change and regional recurrence among treatment groups (department A: multiple-step action since October 2015) and control group (department B: without implementation of multiple-step action) [14, 15]. A two-sided test with a *p* value of < 0.05 represented statistical significance.

## Results

After propensity score matching, 400 patients were enrolled for analysis. The mean patient age was 55.2 ± 11.1 years and 92% were male (Table 1). In period 1, patients treated by department A surgeons were more likely to be older, have tongue cancer, and received adjuvant radiotherapy. In the period after October 2015, patients treated by department A surgeons were more likely to have tongue cancer.

**Table 1:** Demographic and clinical characteristics of study patients, *n* = 400

	Period 1 (01–2009–09–2015)			Period 2 (10–2015–09–2018)		
Variable	Department A (n = 118)	Department B (n = 118)	*p* value	Department A (n = 82)	Department B (n = 82)	*p* value
Gender			0.232			0.773
Male	106(89.8)	111(94.1)		75(91.5)	76(92.7)	
Female	12(10.2)	7(5.9)		7(8.5)	6(7.3)	
Age (Mean ± SD)	56.4 ± 11.2	52.7 ± 10.3	0.008	55.5 ± 10.0	55.3 ± 12.0	0.916
cN classification			0.091			0.073
N0	57(48.3)	62(52.5)		45(55.6)	60(73.2)	
N1	20(16.9)	9(7.6)		10(12.3)	8(9.8)	
N2	41(34.7)	47(39.8)		26(31.7)	14(17.1)	
N3	0(0.0)	0(0.0)		1(0.9)	0(0.0)	
cT classification			0.152			0.148
T1	26(22.0)	19(16.1)		16(19.8)	21(25.6)	
T2	46(39.0)	42(35.6)		28(34.1)	22(26.8)	
T3	7(5.9)	3(2.5)		12(14.8)	5(6.1)	
T4	39(33.1)	54(45.8)		26(32.1)	34(41.5)	
Differentiation			0.085			0.682
Well	17(14.4)	27(22.9)		14(17.1)	14(17.1)	
Moderately	91(77.1)	87(73.7)		60(73.2)	63(76.8)	
Poorly	10(8.5)	4(3.4)		8(9.8)	5(6.1)	
Tumor subsite			< 0.001			0.002
Tongue	63(53.4)	22(18.6)		36(43.9)	18(22.0)	
Buccal	40(33.9)	55(46.6)		32(39)	33(40.2)	
Other sites	15(12.7)	41(34.7)		14(17.1)	31(37.8)	
Margin			0.651			0.096
Negative	116(98.3)	115(97.5)		77(93.9)	81(98.7)	
Positive	2(1.7)	3(2.5)		5(6.1)	1(1.2)	
Adjuvant treatment			0.002			0.064
Nil	56(47.5)	54(45.8)		35(42.7)	50(61.0)	
RT	47(39.8)	29(24.6)		36(43.9)	23(28.0)	
CT	2(1.7)	14(11.9)		0(0.0)	0(0.0)	
RT + CT	13(11.0)	21(17.8)		11(13.4)	9(11.0)	

**Fig. 2:**
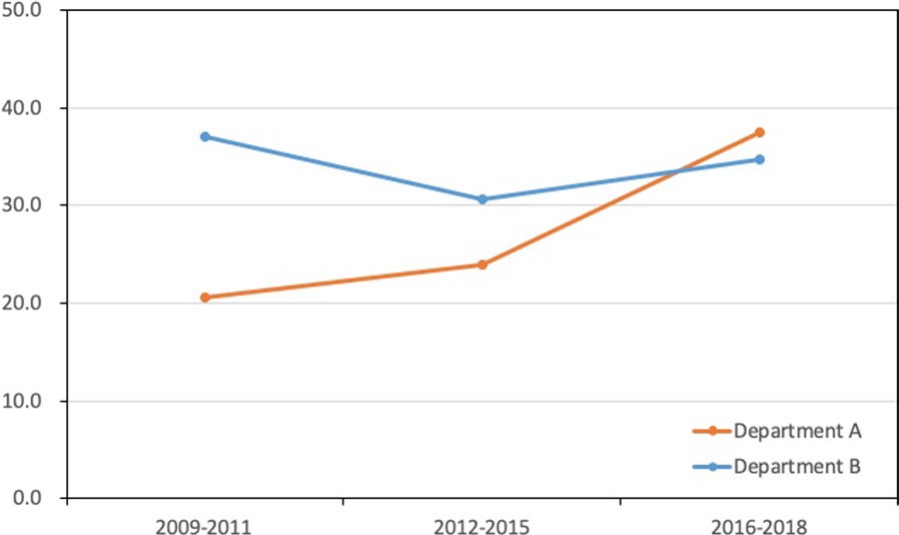
Mean lymph node yield in different groups and periods

The average LN yield by the two different departments is illustrated in Fig. 2 and Additional file 1: Table S1. As shown in Table 2, compared with the control group in period 1, the average LN yield was lower in department A (22.8 vs 33.4, *p* < 0.001). In period 2 the average LN yield was similar between department A and department B (37.4 vs 33.9, *p* = 0.099). This finding suggests there was a significant increase in LN yield in department A after October 2015, however the LN yield in department B was not statistically different. During the period before October 2015, there was no significant difference in regional recurrence between the two groups (8.5% vs 5.9%, *p* = 0.45; Table 3). In the period after October 2015, regional recurrence was lower, however no significant differences between two groups were observed (2.5% vs 3.7%, *p* = 0.65).

**Table 2:** Mean values of lymph node yield by different departments and periods

Variable	Period 1 (01–2009–09–2015)	Period 2 (10–2015–09–2018)	Difference**	*p* value^#^
All patients				
Department A	22.8	37.4	14.6	< 0.001
Department B	33.4	33.9	0.5	0.800
Difference*	− 10.6	− 3.5		
* p* value^#^	< 0.001	0.099		
END				
Department A	21.2	34.2	13.0	< 0.001
Department B	29.3	33.5	4.2	0.053
Difference*	8.1	0.7		
* p* value^#^	< 0.001	0729		
RND				
Department A	24.3	41.5	17.2	< 0.001
Department B	38.0	35.0	− 3.0	0.395
Difference*	13.1	6.3		
* p* value^#^	< 0.001	0.110		

END, elective neck dissection; RND, radical neck dissection

^*^Difference between Department A group and Department B group

^**^Difference between period 1 and period 2

^#^Two-sample t-test

**Table 3:** Regional recurrence rate by different departments and periods

Variable	Period 1 (01–2009–09–2015)	Period 2 (10–2015–09–2018)	Difference**	*p* value^#^
All patients				
Department A	10/118(8.5%)	2/81(2.5%)	− 6.0	0.080
Department B	7/118(5.9%)	3/82(3.7%)	− 2.2	0.468
Difference*	2.6	− 1.2		
* p* value^#^	0.450	0.650		
END				
Department A	6/57(10.5%)	2/45(4.4%)	− 6.1	0.257
Department B	0/62(0.0)	2/60(3.3%)	3.3	0.147
Difference*	10.5	1.1		
* p* value^#^	0.010	1.000		
RND				
Department A	4/61(5.6%)	0/36(0.0)	− 5.6	0.117
Department B	7/56(12.5%)	1/22(4.6%)	− 7.9	0.297
Difference*	− 6.9	− 4.6		
* p* value^#^	0.348	0.373		

END, elective neck dissection; RND, radical neck dissection

^*^Difference between Department A group and Department B group

^**^Difference between period 1 and period 2

^#^Pearson’s chi-square test or Fisher’s exact test

In multivariate analysis, LN yield in department A in period 2 was the highest (beta coefficient = 15.6; 95% CI 12.17–19.03) (Table 4). Regional recurrence in department A in period 2 was the lowest, however a statistical difference was not obtained (OR 0.21; 95% CI 0.003–1.74). We also used the difference-in-difference analysis to estimate the impact of multiple-step action on LN yield and regional recurrence. The net impact, with a coefficient of 13.78 (*p* < 0.001) represented the improvement of LN yield in department A after launching the multiple-step action plan in October 2015 (Table 5). A borderline protective effect of the multiple-step action plan on LN yield for cN0 patients was demonstrated with a reduced regional recurrence rate of 11.6% (*p* = 0.072).

**Table 4:** Multivariate analysis for lymph node yield and regional recurrence

	Lymph node yield			Regional recurrence		
	All patient	END	RND	All patient	END	RND
Group						
Department A in Period 1	Ref	Ref	Ref	Ref	Ref	Ref
Department B in Period 1	12.09 (8.86–15.32)	9.09 (4.95–13.23)	16.07 (10.73–21.42)	0.57 (0.20–1.69)	–	2.29 (0.45–11.69)
Department A in Period 2	15.60 (12.17–19.03)	13.52 (8.97–18.06)	18.64 (13.10–24.18)	0.21 (0.03–1.74)	0.43 (0.05–4.17)	–
Department B in Period 2	13.91 (10.40–17.43)	12.93 (8.77–17.10)	15.46 (8.65–22.28)	0.30 (0.06–1.49)	0.37 (0.07–2.01)	–
Female	1.35 (− 3.08–5.78)	1.23 (− 4.74–7.21)	1.94 (− 5.02–8.91)	0.81 (0.10–6.63)	–	2.28 (0.19–26.72)
Age	− 0.22 (− 0.33 to − 0.11)	− 0.14 (− 0.28–0.00)	− 0.32 (− 0.50 to − 0.13)	0.99 (0.94–1.03)	1.01 (0.94–1.08)	0.97 (0.90–1.04)
cT3-4	1.29 (− 1.24–3.82)	0.08 (− 3.21–3.37)	2.03 (− 2.06–6.13)	1.14 (0.41–3.15)	0.48 (0.07–3.24)	1.53 (0.37–6.24)
cN2-3	7.37 (4.67–10.06)		7.54 (2.93–12.15)	1.19 (0.43–3.30)	NA	0.59 (0.12–2.84)
Differentiation: poor	0.97 (− 3.81–5.74)	− 2.06 (− 9.49–5.36)	3.44 (− 3.16–10.04)	–	–	–
None tongue	− 3.05 (− 5.75 to − 0.34)	− 2.08 (− 5.58–1.43)	− 5.07 (− 9.53 to − 0.62)	1.45 (0.49–4.30)	2.58 (0.44–15.21)	0.85 (0.17–4.24)
Margin: positive				–	–	–
With adjuvant treatment				1.37 (0.49–3.84)	2.31 (0.38–13.82)	1.19 (0.28–4.97)

**Table 5:** Difference-in-differences analysis for lymph node yield and regional recurrence

	Lymph node yield		Regional recurrence	
	Coefficient*	*p* value	Coefficient** (%)	*p* value
All patient				
Time effect	1.82	0.291	− 2.4	0.488
Difference-in-difference (net) impact	13.78	< 0.001	− 3.9	0.448
END				
Time effect	3.84	0.057	5.1	0.205
Difference-in-difference (net) impact	9.68	0.001	− 11.6	0.072
RND				
Time effect	− 0.61	0.849	− 11.7	0.081
Difference-in-difference (net) impact	19.25	< 0.001	5.6	0.534

END, elective neck dissection; RND, radical neck dissection

^*^Regressions controlled for sex, age, differentiation, tumor sub-site (tongue vs non-tongue), cT, and cN

^**^Regressions controlled for sex, age, cT, cN, differentiation, tumor sub-site (tongue vs non-tongue), margin status, and adjuvant treatment modality (without vs with adjuvant treatment)

## Discussion

Although there have been several breakthrough innovations in the treatment of oral cancer (e.g., target therapy or immune checkpoint inhibitors), patients with oral cancer with regional recurrence frequently incur grave outcomes [2–4, 6]. Besides radical resection of primary tumor with adequate margin, the next step was to perform an adequate neck dissection. Previous studies have reported a positive association between LN yield and survival rates [11, 18]. However, strategies to improve LN yield have not been identified. The current study was the first to explore the impact of non-compulsory multiple-step action among surgeons to improve the quality of neck dissection and several novel findings were noted. First, the difference in the difference was an increase of LN yield of 13.78. Second, a reduction of 11.6% in regional recurrence was noted in cN0 oral cancer patients (Table 5). These findings have relevance to long-term outcomes in head and neck cancers. Government agencies, like the health promotion administration in Taiwan, regularly announced the survival rates of major cancers. However, strategies to improve outcomes are not clearly outlined. Our results provide evidences about that multiple-step action was associated with increased LN yield and decreased regional recurrence in patients with oral cancer. This observed activity may promote surgeons to improve the quality of neck dissections, is simple for clinical use.

Several mechanisms might explain the effect of the multiple-step action plan. First, landmark identification during neck dissection was not emphasized purposely in department A. Our previous publication and related literature outlined the importance of maximization of LN yield, and the best and simplest strategy was to perform an en-bloc neck dissection with landmark identifications in order to preserve function and maximize the LN yield simultaneously [11, 13]. Second, the positive association between LN yield and survival rates was stressed routinely in weekly conferences in department A. Understanding mapping between the LN yield and outcomes urged the staff to perform a neck dissection to increase the LN yield in the hope to decrease regional recurrences later. Third, the average number of the LN yield in department A was announced regularly, and the individual data was not reported. According to behavior economics, the staff with lower LN yield compared with the average yield from all staff started to figure out how to improve LN yield [19]. Beyond surgical techniques and pathological analysis, increased LN yield was associated with an average delay in surgery over 15 days, skin involvement by tumor, and additional precancer lesions in oral cancer [20]. In our series, there was no significant difference in age, gender, and differentiation between groups. In the multivariate analysis, the above-mentioned symptoms were adjusted in the regression model.

The magnitude of the 64% increase in LN yield and decrease of regional recurrence in department A after implementation of the multiple-step action plan was significant (Tables 2 and 3). Increase of LN yield and decrease in regional recurrence in department A with multi-step action plan was summarized in Table 4. Besides multivariate linear and logistic regression, the impact of the multiple-step action plan was also evaluated with difference-in-difference analysis. The major policy change in department A over time was the intervention or policy change in period 2, which provided for a quasi-experimental chance. Using difference-in-difference analysis, the net effect or interaction term, intervention × period on LN yield and regional recurrence was evaluated with the linear model. The multiple-step action plan incurred a net impact of 13.78 increase in LN yield and a reduction of 11.6% in regional recurrence in the END group. We also analyzed the overall survival rates between different groups and periods (Additional file 2: Figure S1). Patients with local or loco-regional recurrence were excluded. There was no significant difference among these groups, which might be attributed to different follow-up periods and heterogenous surgical techniques.

All national and international guidelines have reported how adequate LN yield could significantly improve survival in patients with oral cancer, however there is no recommended intervention for clinical practice. Interventions based on the theories of behavior economics are summarized by the acronym NUDGE and has been explored in the healthcare field [21–23]. Penn Medicine, for example, has used this strategy widely. Changing the default choice for medications greatly increased the rate of prescriptions for generic medicine [22]. Using an active choice alert system in the electric medical record increased the influenza vaccination rate 37% in adults suitable for vaccination [24]. Ayala et al. also reported that moving from an "opt-in" to an "opt-out" system greatly increased the rate of providing aspirin prophylaxis for preeclampsia prevention [25]. An automated dashboard with active choices and peer comparison performance feedback to physicians was associated with increased statin prescriptions by primary care physicians [26]. In our institute, several feasible and low-cost strategies have been applied, such as weekly conferences used to explain the association between LN yield and recurrence since October 2015. Thereafter, surgeons were encouraged to confirm important landmarks during neck dissections. Furthermore, the average LN yield was announced each month in order to provide feedback to all surgeons. Multiple NUDGE-like interventions helped the target group to improve the cervical LN yield in oral cancer surgery.

There are several limitations in the current study. First, the study was not a prospective design. All these data were obtained retrospectively. Second, the common trend assumption was not well tested in our series for DID analysis. Third, we could only confirm that surgery oriented by landmarks was emphasized in department A during period 2; however, it could be a standard operative procedure in department B. Fourth, the spillover effects of the multiple-step action plan in the control group was not estimated. Although it was possible, the effect may be minimal because these two departments were located in different floors in our institute. Fifth, the time effect of diagnosis may also influence the LN yield. The estimated LN yield could be derived from the data between 2009 and 2015 (Additional file 1: Table S2). The gap between the real LN yield (37.5) and estimated LN yield (27.4) could be attributed to the multi-step intervention in department A. Furthermore, the annual LN increase was 0.4 since 2009 in SEER database (data not shown). The increase in LN yield was much than the annual increase by time. Finally, although this study only included Asian people, this observed activity improving the quality of neck dissections, is simple, and could be applied to a widespread patient population.

## Conclusions

Previous literature has validated the importance of LN yield in head and neck cancer. Interventions such as multiple-step action used in this study was associated with increased LN yield and decreased regional recurrence in patients with oral cancer undergoing neck dissection. This soft and non-mandatory practice could be applied across institutions in order to improve the quality of oral cancer treatment and survival rates.

## Supplementary information

**Additional file 1: Table S1.** Mean lymph node yield in different groups and periods. **Table S2**. Difference between real LN yield and estimated number from liner regression.

**Additional file 2: Figure S1.** The overall survival rates between different groups and periods.

## Data Availability

The datasets used during the present study are available from the corresponding author upon reasonable request.

## Abbreviations

LN: Lymph node
END: Elective neck dissection
RND: Radical neck dissection
DID: Difference-in-difference
AJCC: American Joint Committee on Cancer
HR: Hazard ratio
CI: Confidence interval

## References

[1] Siegel RL, Miller KD, Jemal A (2019). Cancer statistics, 2019. CA Cancer J Clin.

[2] Rogers SN, Brown JS, Woolgar JA, Lowe D, Magennis P, Shaw RJ, Sutton D, Errington D, Vaughan D (2009). Survival following primary surgery for oral cancer. Oral Oncol.

[3] Ferlazzo N, Curro M, Zinellu A, Caccamo D, Isola G, Ventura V, Carru C, Matarese G, Ientile R (2017). Influence of MTHFR genetic background on p16 and MGMT methylation in oral squamous cell cancer. Int J Mol Sci.

[4] Isola G, Polizzi A, Patini R, Ferlito S, Alibrandi A, Palazzo G (2020). Association among serum and salivary A. actinomycetemcomitans specific immunoglobulin antibodies and periodontitis. BMC Oral Health.

[5] Ettinger KS, Ganry L, Fernandes RP (2019). Oral cavity cancer. Oral Maxillofac Surg Clin N Am.

[6] Woolgar JA, Rogers S, West CR, Errington RD, Brown JS, Vaughan ED (1999). Survival and patterns of recurrence in 200 oral cancer patients treated by radical surgery and neck dissection. Oral Oncol.

[7] Visser E, Markar SR, Ruurda JP, Hanna GB, van Hillegersberg R (2019). Prognostic value of lymph node yield on overall survival in esophageal cancer patients: a systematic review and meta-analysis. Ann Surg.

[8] Lykke J, Rosenberg J, Jess P, Roikjaer O (2019). Danish Colorectal Cancer G: Lymph node yield and tumour subsite are associated with survival in stage I-III colon cancer: results from a national cohort study. World J Surg Oncol.

[9] Kuo P, Mehra S, Sosa JA, Roman SA, Husain ZA, Burtness BA, Tate JP, Yarbrough WG, Judson BL (2016). Proposing prognostic thresholds for lymph node yield in clinically lymph node-negative and lymph node-positive cancers of the oral cavity. Cancer.

[10] Lee CC, Lin YS, Kang BH, Chang KP, Chi CC, Lin MY, Su HH, Chang TS, Chen HC, Chen PC (2017). Incorporation of log odds of positive lymph nodes into the AJCC TNM classification improves prediction of survival in oral cancer. Clin Otolaryngol.

[11] Divi V, Chen MM, Nussenbaum B, Rhoads KF, Sirjani DB, Holsinger FC, Shah JL, Hara W (2016). Lymph node count from neck dissection predicts mortality in head and neck cancer. J Clin Oncol.

[12] Ebrahimi A, Clark JR, Zhang WJ, Elliott MS, Gao K, Milross CG, Shannon KF (2011). Lymph node ratio as an independent prognostic factor in oral squamous cell carcinoma. Head Neck.

[13] Lee CC, Su YC, Hung SK, Chen PC, Huang CI, Huang WL, Lin YW, Yang CC (2017). Recommendation for incorporation of a different lymph node scoring system in future AJCC N category for oral cancer. Sci Rep.

[14] Carey K, Lin MY (2015). Readmissions to New York hospitals fell for three target conditions from 2008 to 2012, consistent with medicare goals. Health Aff (Millwood).

[15] Raifman J, Moscoe E, Austin SB, Hatzenbuehler ML, Galea S (2018). Association of state laws permitting denial of services to same-sex couples with mental distress in sexual minority adults: a difference-in-difference-in-differences analysis. JAMA Psychiatry.

[16] Wing C, Simon K, Bello-Gomez RA (2018). Designing difference in difference studies: best practices for public health policy research. Annu Rev Public Health.

[17] Fernandes R, Ord R (2006). Access surgery for oral cancer. Oral Maxillofac Surg Clin North Am.

[18] Cramer JD, Speedy SE, Ferris RL, Rademaker AW, Patel UA, Samant S (2017). National evaluation of multidisciplinary quality metrics for head and neck cancer. Cancer.

[19] Patel MS (2018). Nudges for influenza vaccination. Nat Hum Behav.

[20] Muttagi SS, Patil BR, Godhi AS, Arora DK, Hallikerimath SR, Kale AD (2016). Clinico-pathological factors affecting lymph node yield in Indian patients with locally advanced squamous cell carcinoma of mandibular Gingivo-Buccal sulcus. Indian J Cancer.

[21] Binns C, Low WY (2017). Nobel prizes, nudge theory, and public health. Asia Pac J Public Health.

[22] Bourdeaux CP, Davies KJ, Thomas MJ, Bewley JS, Gould TH (2014). Using 'nudge' principles for order set design: a before and after evaluation of an electronic prescribing template in critical care. BMJ Qual Saf.

[23] O'Reilly-Shah VN, Easton GS, Jabaley CS, Lynde GC (2018). Variable effectiveness of stepwise implementation of nudge-type interventions to improve provider compliance with intraoperative low tidal volume ventilation. BMJ Qual Saf.

[24] Patel MS, Volpp KG, Small DS, Wynne C, Zhu J, Yang L, Honeywell S, Day SC (2017). Using active choice within the electronic health record to increase influenza vaccination rates. J Gen Intern Med.

[25] Ayala NK, Rouse DJ (2019). A nudge toward universal aspirin for preeclampsia prevention. Obstet Gynecol.

[26] Patel MS, Kurtzman GW, Kannan S, Small DS, Morris A, Honeywell S, Leri D, Rareshide CAL, Day SC, Mahoney KB (2018). Effect of an automated patient dashboard using active choice and peer comparison performance feedback to physicians on statin prescribing: the PRESCRIBE cluster randomized clinical trial. JAMA Netw Open.

