# Supplementary material for: Association of a multiple-step action with cervical lymph node yield of oral cancer patients in an Asian country
Source: BMC Oral Health. 2021 Jan 13;21:29. doi: 10.1186/s12903-021-01389-3 (PMC7805045; doi:10.1186/s12903-021-01389-3)
Supplement: Supplementary file 1 — Additional file 1: Table S1. Mean lymph node yield in different groups and periods. Table S2. Difference between real LN yield and estimated number from liner regression. [file 12903_2021_1389_MOESM1_ESM.docx]

Additional file 1: Table S1. Mean lymph node yield in different groups and periods.

| Period | Department A | (95% C.I.) | Department B | (95% C.I.) |
| --- | --- | --- | --- | --- |
| 2009-2011 | 20.6 | (16.7-24.5) | 37.0 | (32.7-41.3) |
| 2012-2015 | 24.0 | (21.6-26.3) | 30.6 | (28.1-33.2) |
| 2016-2018 | 37.5 | (34.3-40.7) | 34.7 | (31.7-37.6) |

| Additional file 1: Table S2. Difference between real LN yield and estimated number from liner regression. | | | |
| --- | --- | --- | --- |
|  | Estimation equation derived form 2009-2015 | Estimated LN yield between 2016-2018 | Real LN yield between 2016-2018 |
| All pts | Y=30.8-3.8X | 23.2 | 36.1 |
| Department A | Y=20.6+3.4X | 27.4 | 37.5 |
| Department B | Y=37.0-6.4X | 24.2 | 34.7 |
