# Supplementary material for: Association of a multiple-step action with cervical lymph node yield of oral cancer patients in an Asian country
Source: BMC Oral Health. 2021 Jan 13;21:29. doi: 10.1186/s12903-021-01389-3 (PMC7805045; doi:10.1186/s12903-021-01389-3)
Supplement: Supplementary file 2 — Additional file 2: Figure S1. The overall survival rates between different groups and periods. [file 12903_2021_1389_MOESM2_ESM.docx]

| Additional file 2: Figure S1. The overall survival rates between different groups and periods. | | |
| --- | --- | --- |
| All patients | With elective neck dissection | With radical neck dissection |
| 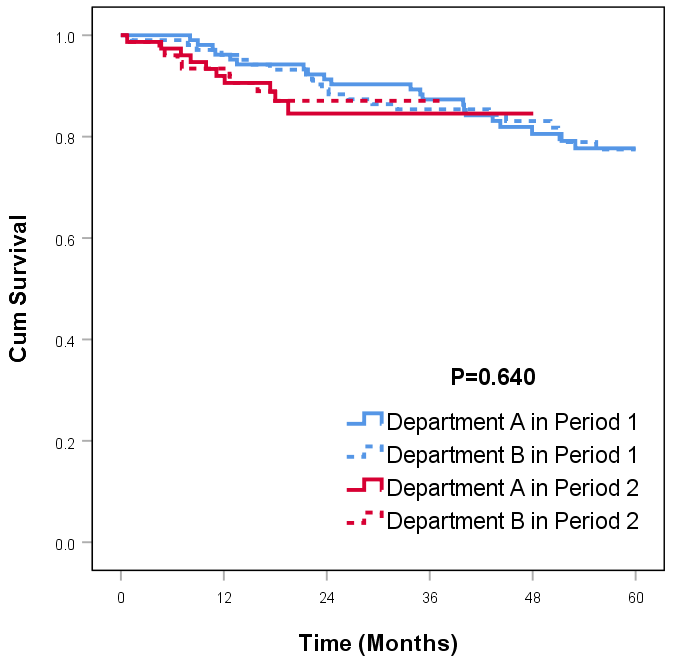 | 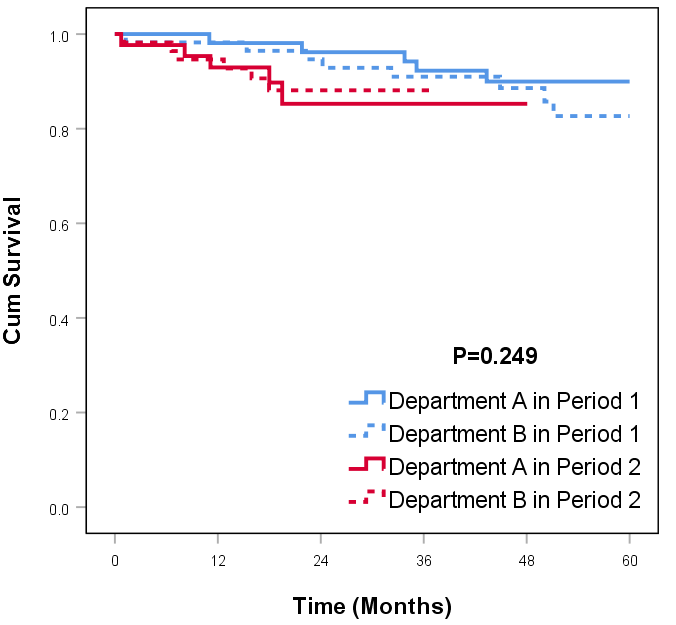 | 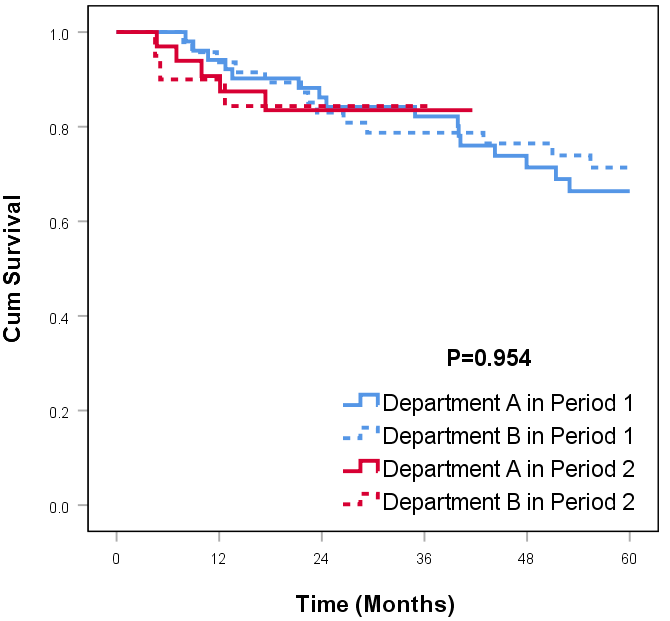 |
